# TB and COVID-19: An Exploration of the Characteristics and Resulting Complications of Co-infection

**DOI:** 10.31083/j.fbs1401006

**Published:** 2022-03-01

**Authors:** Erica Luke, Kimberly Swafford, Gabriella Shirazi, Vishwanath Venketaraman

**Affiliations:** 1 College of Osteopathic Medicine of the Pacific, Western University of Health Sciences, Pomona, CA 91766-1854, USA

**Keywords:** COVID-19, latent TB, active TB, tuberculosis, SARS, MERS, immune response, anti-inflammatory agents, epidemiology, bronchiectasis, fibrosis, cavitation, TB treatment, IL-10, TNF-*α*, IFN, TGF-*β*, IL-35, Regulatory T cells, co-infection

## Abstract

Tuberculosis (TB) and Coronavirus Disease-19 (COVID-19) infection are two respiratory diseases that are of particular concern epidemiologically. Tuberculosis is one of the oldest diseases recorded in the history of mankind dating back thousands of years. It is estimated that approximately one quarter of the world’s population is infected with latent Mycobacterium tuberculosis (LTBI). This contrasts with COVID-19, which emerged in late 2019. Data continues to accumulate and become available on this pathogen, but the long-term side effect of fibrotic damage in COVID-19 patients evokes parallels between this novel coronavirus and its ancient bacterial affiliate. This similarity as well as several others may incite inquiries on whether coinfection of individuals with latent TB and severe acute respiratory syndrome coronavirus 2 (SARS-CoV-2) lead to excessive fibrosis in the lungs and thus the emergence of an active TB infection. While it is well understood how TB leads to structural and immunological lung complications including granuloma formation, fibrosis, and T cell exhaustion, less is known about the disease course when coinfection with SARS-CoV-2 is present. Past and present research demonstrate that IL-10, TNF-*α*, IFN class I-III, TGF-*β*, IL-35, and Regulatory T cells (T-regs) are all important contributors of the characteristics of host response to mycobacterium tuberculosis. It has also been noted with current research that IL-10, TNF-*α*, IFN class I, II, and III, TGF-*β*, ACE-2, and T-regs are also important contributors to the host response to the SARS-CoV-2 virus in different ways than they are to the TB pathogen. Both pathogens may lead to an unbalanced inflammatory immune response, and together a shared dysregulation of immune response suggests an increased risk of severity and progression of both diseases. We have reviewed 72 different manuscripts between the years 1992 and 2021. The manuscripts pertaining to the SARS-COV-2 virus specifically are from the years 2020 and 2021. Our literature review aims to explore the biomolecular effects of these contributors to pathogenicity of both diseases along with current publications on TB/COVID-19 coinfection, focusing on the pathogenicity of SARS-CoV-2 infection with both latent and active TB, as well as the challenges in treating TB during the COVID-19 pandemic. The compiled material will then aid the latticework foundation of knowledge for future research leading to a hopeful improved system of therapeutic strategies for coinfection.

## 1. Introduction

Tuberculosis (TB) is caused by the bacterium *Mycobacterium tuberculosis* (Mtb), a highly aerobic bacillus that most commonly colonizes in the lungs. Approximately a quarter of the world’s population is latently infected with tuberculosis. It is one of the top 10 causes of death worldwide and the number one cause of infectious disease deaths worldwide with a rising mortality [1]. Documented incidence in 2020 showed a 20% decrease in TB diagnoses, which is suspected to be the product of many factors. Some of these factors may include reduced access to diagnostic, treatment and preventative services, and misdiagnosis due to the Coronavirus Disease 19 (COVID-19) pandemic [2], as well as a global increase in mask wearing and social distancing. The World Health Organization (WHO) Global Tuberculosis Report 2021 has speculated that the COVID-19 pandemic has reversed years of progress in the effort to globally eradicate TB. The recorded TB diagnosis dropped from 7.1 million in 2019 to 5.8 million in 2020, but it is estimated that approximately 10 million people newly acquired TB in 2020. Drug resistant TB continues to be a threat, making up a statistically significant number of cases. For instance, 132,222 Multidrug- and rifampicin-resistant tuberculosis (MDR/RR-TB) and 25,681 Extensive and Pre-Extensively drug-resistant tuberculosis (XDR-TB, pre-XDR-TB) were reported globally in 2020, and 150,359 people in total were enrolled in MDR/RR-TB treatment worldwide. This number has decreased 15% from 177,100 in 2019, and it is estimated that only one in three people who develop MDR/RR-TB actually enroll in treatment programs [1].

The COVID-19 pandemic has had significant impacts on global public health and has adversely affected essential healthcare services. The etiology of the COVID-19 pandemic is Severe Acute Respiratory Syndrome Coronavirus 2 (SARS-CoV-2), a rapidly spreading virus which emerged from Wuhan, China in late 2019 resulting in large-scale mortality and morbidity, overwhelming the healthcare system globally [3]. The condition caused by this virus is characterized as a whole spectrum of disease ranging from asymptomatic to rapidly fatal, the severity of which varies greatly based on factors such as viral strain and host comorbidities. As of October 25, 2021, there have been 243,260,214 confirmed cases and 4,941,039 confirmed deaths reported to the WHO. Despite extreme vaccination efforts, the course of the pandemic remains unpredictable, and the challenge to restrain the pandemic is further compounded by the emergence of several SARS-CoV-2 variants viz. B.1.1.7 (Alpha), B.1.351 (Beta), P1 (Gamma) and B.1.617.2 (Delta), which show increased transmissibility and resistance towards vaccines [4]. As of publication of this review, the world remains immersed in the COVID-19 pandemic caused by SARS-CoV-2 while the development of effective antiviral therapies for treating SARS-CoV-2 infection remain in progress [4]. The effort to understand this novel coronavirus has contributed to significant research on the subject, but there is still much to be discovered about the coinfection between this virus and other major morbidities such as tuberculosis. This article explores the characteristics of coinfection using case studies and exploration of the major immune system factors Interleukin-10 (IL-10), Tumor Necrosis Factor Alpha (TNF-*α*), Interferon (IFN) class I-III, Transforming Growth Factor Beta (TGF-*β*), Interleukin-35 (IL-35), Angiotensin Converting Enzyme II (ACE-2) and Regulatory T cells (T-regs) in both disease processes separately and together, respectively. Thus far we have not found a general precedent set on whether any respiratory viral infections consistently cause TB exacerbations. Severe Acute Respiratory Syndrome 1 (SARS-CoV-1) and Influenza type A have been inspected in the past for possible co-infection complications, but studies have yielded mixed or inconclusive results [5–9].

## 2. Host Immune Responses and Pathogenesis of TB

Mtb is phagocytosed by macrophages once in the lungs but is not killed by these immune cells due to its successful blockage of the bridging molecule Early Endosomal Autoantigen 1 (EAA1), which is required for fusion of the phagosome and lysosome during pathogen destruction after phagocytosis [10]. This and several other defense mechanisms prevent the bacterium from destruction and allow unregulated replication inside the macrophage. Cytokines such as Tumor necrosis factor alpha (TNF-*α*), Interferons (IFN), T cells, and upregulation proteins, however, are successful in creating chronic pro-inflammatory responses in the lung parenchyma necessary to maintain granulomas in the lungs that wall off the pathogen to further spread, leading to a “latent” infection [11]. It has been well established that as long as the immune system is adequately maintained, these defenses can keep the bacterium idle indefinitely. The infection process for the host is entirely asymptomatic until these defenses are brought down, and the infection becomes “active” and able to spread throughout the body [12].

TNF*α* is produced by macrophages during the phagocytosis process in response to bacterial proteins. TNF*α* leads to greater production of IL1 and prostaglandin E2 (PGE2), which produce the cardinal symptoms of inflammation (heat, swelling, etc.). TNF*α* inhibitors such as infliximab bind directly to TNF*α*, leading to inflammation attenuation.

The IFN cytokines are key players in many defense processes against infections through the stimulation of hundreds of interferon-stimulated genes (ISGs). The IFN group of cytokines are divided into 3 classes: Class I, II, and III. The class I IFN family is critical in carrying out various immunoregulatory and inflammatory processes. This protein family includes 13 IFN*α* subtypes and 2 IFN*β* subtypes. They are secreted by various cell types, including fibroblasts, T cells, macrophages and NK cells. Their main objective is to produce antiviral and anti-tumor responses. Several IFN*α* subtypes are also pyrogenic and analgesic, through binding to opioid receptors in the brain, causing release of PGE2. The type III IFN group is composed of four IFN-λ molecules called IFN-λ1, IFN-λ2, IFN-λ3 and IFN-λ4. The objective of the type III IFN group is approximately the same as the type I IFN group but less potent and mostly located in the epithelium [13–15].

The class II IFN family has one single member: IFN-*γ*. It is the primary cytokine that defines Th1 cells. It is a dimerized soluble type 2 class of interferon cytokines and an important activator of macrophages. It is produced by NK cells, Th1, and Th8 cells. IFN-*γ* binds to its receptor and activates JAK-STAT pathway, which ultimately leads to an increase in antigen presentation and lysosomal activity on macrophages, as well as increased expression of MHCI and MHCII, leukocyte migration, and primes alveolar macrophages against secondary bacterial infection. In the context of tuberculosis, IFN-*γ* activates macrophages and causes a large intracellular killing response, allowing macrophages to overcome inhibited phagolysosome response caused by mycobacteria [16–18].

**Fig. 1.**
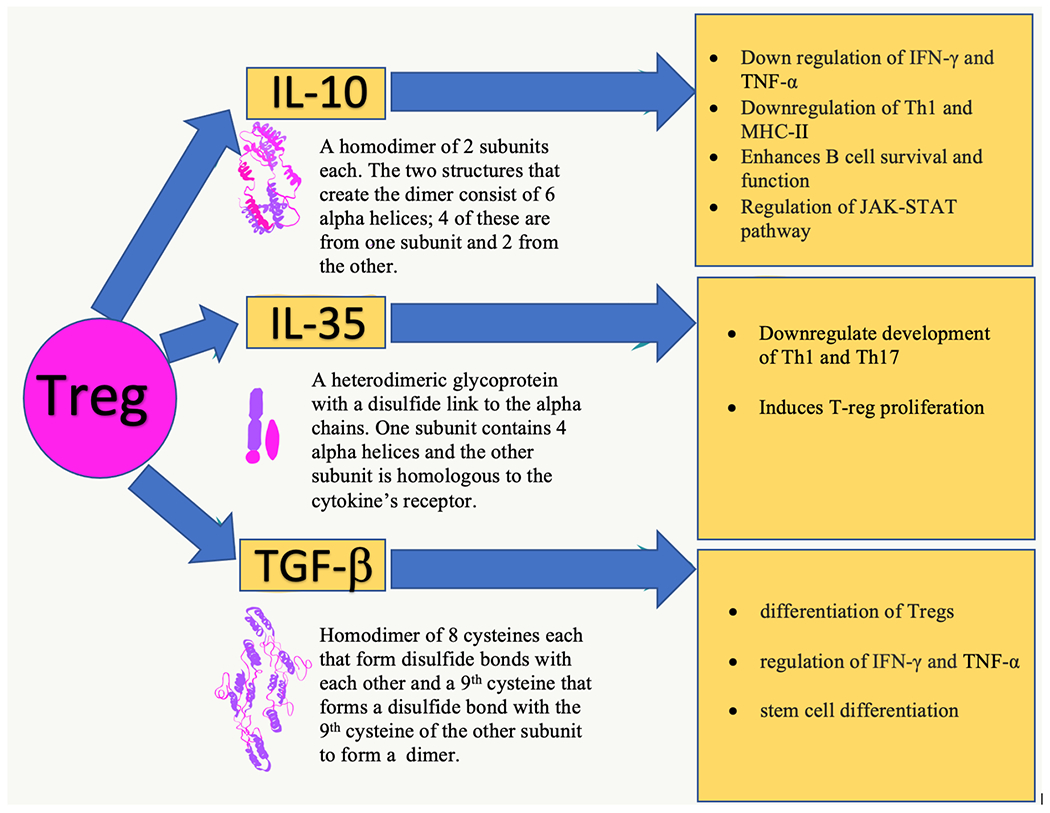
The structures [22–24] and several functions of key cytokines involved in the immunosuppressive function of T-regs.

T-regs, also known as suppressor T cells, are a subdivision of T cells responsible for the recognition of self antigens and the prevention of autoimmune disease. These cells’ control of autoimmunity makes them inherently immunosuppressive [19,20]. In the specific context of TB, T-regs change their role depending on the disease stage. In the early stages of infection, T-regs contribute to immunosuppression, whereas in the chronic stage, T-regs are helpful in counter-regulating excessive inflammation, thereby preventing or delaying disease pathology. T-regs can be broadly classified into Thymic T-regs (tTreg), or Induced T-regs (iTreg). tTregs are produced in the thymus and are responsible for maintaining homeostasis and tolerance systemically. iTregs are formed in the periphery during T cell activation and work locally to dampen antigen-specific immune responses. Inducible T Regulatory Type 1 cells (IT1) are a subdivision of iTregs that use cytokine IL-10 to mediate suppressive effects. The key cytokines involved in the immunosuppressive function of Tregs are IL-10, TGF-*β*, and IL-35. IL-10 and TGF-*β* work to prevent the activity of various cytokines such as IFN-*γ* and TNF-*α*. TGF-*β* works to inhibit the activity of cytotoxic as well as helper T cells. IL-35 induces proliferation of T-reg cells as well as inhibits Th17 activity [21], Fig. 1 (Ref. [22–24]) serves as a guide to the structures and functions of the key cytokines involved in the immunosuppressive function of Tregs.

TGF*β* a multifunctional group of cytokines belonging to the Transforming Growth Factor superfamily of proteins, is critical for the differentiation of T-regs from naive CD4^+^ cells. Its other functions include regulation of inflammatory processes and stem cell differentiation. TB infection has been shown to increase levels of active TGF*β* in the lung. Knowledge of its inflammatory suppression qualities has sparked past research, which has suggested that elimination of TGF*β* signaling may improve T cell function and TB prognosis [25,26]. Studies have shown elevated levels of both TGF*β* and IL-10 in lung samples of primary TB patients through bronchoalveolar lavage [27,28],

The IL-10 family of cytokines has many roles involved with Mtb infection. IL-10, also known as human Cytokine Synthesis Inhibitory Factor (CSIF), can enhance the intracellular survival of mycobacterial bacilli through various mechanisms including: inhibiting phagosomal maturation, reducing nitric oxide production and blocking IFN-*γ* signaling in macrophages [29]. Both human and animal studies have been conducted examining the roles of IL-10 in Mtb and susceptibility to disease with increased levels. Studies in IL-10 transgenic mice have found that IL-10 production can drive TB reactivation and promotion of disease progression [30]. Other rodent studies have examined the effects of blocking or deleting IL-10 function resulting in reduced pulmonary bacterial load and increased resistance to infection through enhanced T cell responses within the lung [31].

Tuberculous granulomas will only form if an engulfing macrophage cannot destroy its phagocytosed foreign material. Macrophages infected by TB that are unable to kill their pathogen release cell signals that cause dendritic cells to recruit Th1 cells, which then become activated and aggregate around macrophages.

**Fig. 2.**
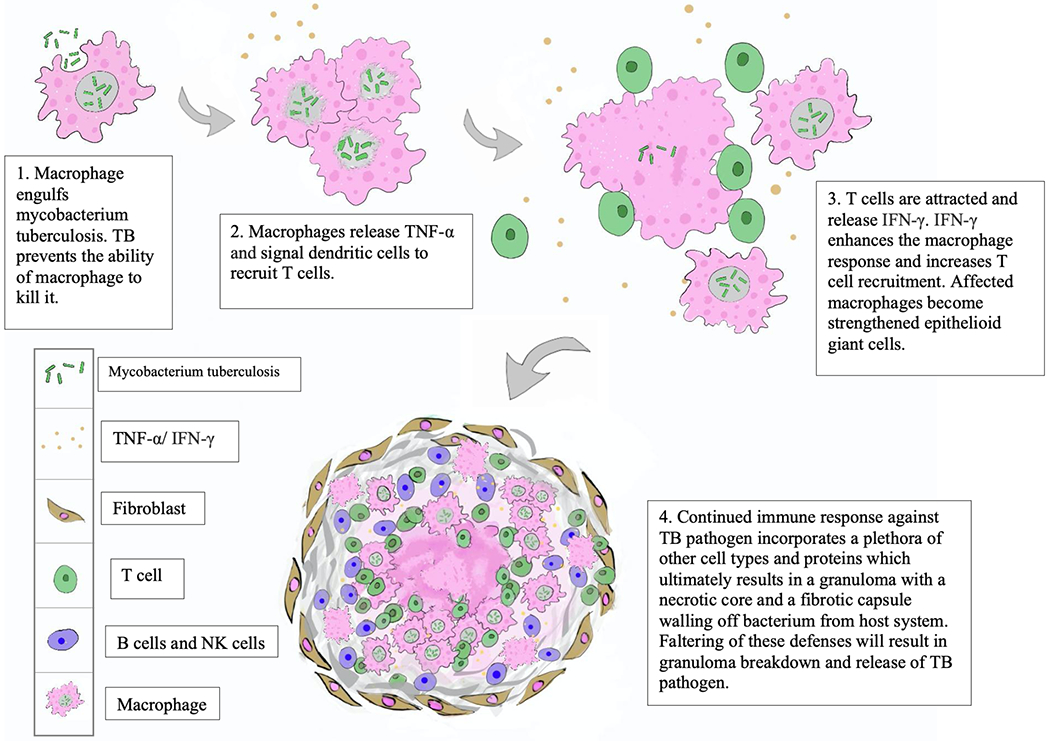
Granuloma formation overview.

Released IFN-*γ* from these Th1 cells enhance the killing response in macrophages and further strengthen the Th1 response. As more macrophages are attracted to the site, they surround the Th1 cells as strengthened epithelioid giant cells and become fibroblast-like, walling off the infection [17,18]. These immune cells are imperative to the maintenance of these granulomas. If these cells or cytokines are compromised, the granulomas usually start to break down, releasing mycobacteria from their confinement. The process of granuloma formation is illustrated in Fig. 2.

## 3. TB Pathology in Autopsy

In post-mortem analysis of a tuberculosis-infected human lung, necrotizing granulomas, or caseum, will be observed. These findings are specific to the tuberculosis pathogen. New caseum reveals a white or yellow cheesy texture. Older caseum becomes grey or chalky. The material is a homogenous, eosinophilic, fine-grained necrosis. Occasionally these granulomas will liquefy and drain, resulting in a cavity. Eventually during the chronic phase of infection, necrotizing granulomas will become invaded with collagenous fibers and solidify into a fibrosed follicle or eventually calcify entirely [32].

## 4. Characteristics of SARS-CoV-2

SARS-CoV-2 is an enveloped, single stranded positive sense RNA virus belonging to the Betacoronavirus genus and has a genome up to 80% similar to SARS-CoV-1 [3,33]. The virion has 4 structural proteins: the Spike (S), Envelope (E), Membrane (M), and Nucleocapsid (N) proteins. The viral S proteins are Type 1 membrane glycoproteins containing a single transmembrane domain oriented extracellularly. It is the numerous copies of S protein oriented extracellularly that give the virus its “crown-like” appearance [34]. For over a decade, it has been known that the Angiotensin Converting Enzyme 2 (ACE2) protein located in humans is a receptor for the S protein in many other members of the Betacoronavirus genus, such as SARS-CoV-1 and MERS-CoV. SARS-CoV-2 is no exception. The S protein in these Betacoronaviridae species is composed of 2 subunits, one of which (S1) binds to ACE2 and the other of which (S2) anchors the S protein to the host cell membrane [34]. ACE2 is a protein belonging to the Angiotensin-Converting Enzyme (ACE) family of dipeptidyl carboxydipeptidases. ACE converts Angiotensin I into vasoconstricting Angiotensin II, and ACE2 converts Angiotensin II into vasodilating Angiotensin 1-7 [35]. ACE2 is variably expressed in multiple organs, such as the kidneys and lungs. While during COVID-19 infection it is a gateway to viral entry into the cell, it is also ironically an important protein in attenuating inflammatory lung disease [36]. The immunomodulators TNF-*α* and TGF*β* also play a key role in the response against the virus; these cytokines are also key inflammation agents in the pro-inflammatory response against the TB bacillus [11].

In general, T-regs are the first line of defense against unregulated inflammatory response during a COVID-19 infection [37]. They are key in manipulating the numbers and expressions of other immune cells in order to maintain self tolerance and immune homeostasis. However, there has been a widely observed variation in T-reg numbers as various studies on the subject have observed that T-reg numbers can be remarkably lower than normal [38,39] or higher than normal [40]. The lack of consistency in studies suggests that more research is necessary to establish a more substantial conclusion on the correlation between T-reg levels and severity of COVID-19 infection.

Many studies have revealed that patients with severe COVID-19 infections have higher levels of cytokines (TNF-*α*, IFN-*γ*, IL-2, IL-4, IL-6 and IL-10) than control individuals, especially during the characteristic COVID-19 cytokine storm. It has been suggested that the consistent correlation between disease severity and levels of IL-6 and IL-10 points to these cytokines as reliable fast diagnostic predictors in disease severity [38,41,42]. This is an interesting observation since IL-6 and IL-10 are contradicting cytokines on the inflammation spectrum. IL-6 is known to be pro-inflammatory and IL-10 is known to be an anti-inflammatory cytokine.

It has been proposed that TGF-*β* is a significant contributor to the characteristics of short term and long term COVID-19 infection. A 2020 article that explores the characteristics of multiorgan damage in infected patients confidently pointed this out due to previous research done on the SARS-CoV-1 epidemic in 2003 and the similar effects that TGF*β* has during these infections. Once these viruses attach to the ACE2 receptor in lung epithelium, internalization and replication of viral materials are undergone. This triggers production of TGF*β* which in turn activates apoptosis, inflammation, and lung fibrosis [43]. The fibrosis in the lungs during COVID-19 infection have been mainly attributed to TGF*β* according to one article that has delved into the subject; it has been proposed that blockade of TGF*β* function may reveal significant improvement in morbidity and mortality [44]. It is important to note the difference between the actions of mild/moderate levels of TGF*β*, which produce an anti-inflammatory effect, and overexpression of TGF*β*, which has fibrotic and damaging effects on lung parenchyma.

Class I and III interferons have a critical role in the defense against viral infections. When Pattern Recognition Receptors (PRR) on immune cells recognize Pathogen Associated Molecular Proteins (PAMPs) affiliated with many viral infections, IFN cytokines will be secreted. Interferons are critical for both the innate and adaptive immune responses because once they are activated, they in turn will activate hundreds of different interferon-stimulated genes (ISGs) that increase the immune response against viral infections [14]. Class I IFNs were first discovered through recognition of their strong response against the influenza virus, and the numerous signaling pathways that IFN cytokines trigger continue to be studied today. The viral inhibition mechanisms are extremely diverse and include mechanisms that target the viral life cycle at almost every step. Some ISGs operate as viral sensors (RIG-I, PKR, IFI16, cGAS), some deter viral entry (IFITM), some impede viral genome replication (Mx), and some block viral release and dissemination (BST-2/CD137) [45].

Addionally, interferons have been implicated in the cytokine storm that affects the host’s system during SARS-CoV-2 infection. SARS-CoV-2 infection produces the increase of inflammatory molecules such as IL-6 and IFN-*γ*, which then activate the JAK/STAT pathway and induce NF-kB signaling. This causes nuclear translocation of NF-kB and p38 phosphorylation, which induces inflammatory cytokines and chemokines that ultimately lead to a cytokine storm [42].

## 5. Pathology in COVID-19 Autopsy

In post-Mortem analysis of a COVID-19-infected human lung, the major histopathological finding is disseminated Diffuse Alveolar Damage (DAD) at different stages, detectable in all lung lobes but not homogeneously effected. The middle and lower lung fields are most heavily affected. Characteristics of DAD include fibroblastic proliferation, fibrosis, and pneumocyte hyperplasia leading to alveolar collapse [46]. Other non-universal findings that may be observed include signs of pulmonary embolism, alveolar hemorrhage, and bronchopneumonia with or without typical features of DAD [47].

## 6. Effect of Covid on Latent TB

Co-infection with latent TB and COVID-19 is of particular concern for various reasons, including potential for missed diagnosis due to nonspecific overlapping clinical features, potential for drug-drug interactions, and increased severity of disease with post-TB sequelae [48]. Our review aimed to analyze current literature on whether COVID-19 may reactivate latent TB. There is emerging evidence that patients with Latent Mycobacterium Tuberculosis Infection (LMTBI) and TB disease have an increased risk of the SARS-CoV-2 infection and predisposition towards developing severe COVID-19 pneumonia, although evidence is not conclusive [49]. In a study by Petrone *et al.* [50], it was demonstrated that COVID-19 patients, either TB or LMTBI, have a low ability to build an immune response to SARS-CoV-2 while retaining the ability to respond to Mtb-specific antigens. Another study reported that long term impact of COVID-19 may activate latent TB post-pandemic, given the history of tuberculosis as a key bacterial infection in past viral pandemics [51].

In the first published cohort study analyzing co-infection of TB and COVID-19 across 8 countries, 49 cases of co-infection were explored and it was found that a diagnosis of COVID-19 may occur before, simultaneously, or after a diagnosis of tuberculosis [52] A case report of a 40 year old female diagnosed with COVID-19 and TB suggested that CD4^+^ T-cell depletion associated with COVID-19 may be implicated in the progression of LMTBI to active TB in a similar manner to Human Immunodeficiency Virus (HIV) [53]. The patient presented 7 weeks after a COVID-19 diagnosis with right sided pleuritic chest pain, cough, subjective fever, and anorexia. The patient reported contact with TB two years prior and sputum for Mtb Polymerase Chain Reaction (PCR) and sputum on Acid-Fast Bacilli (AFB) culture were positive.

In a retrospective analysis by Diao *et al.* [38], the number of total T cells, CD4^+^ and CD8^+^ T cells were significantly reduced in COVID-19 patients, especially in patients requiring Intensive Care Unit (ICU) care. Counts of total T cells, CD8^+^ T cells or CD4^+^ T cells lower than 800, 300, or 400/*μ*L, respectively, were negatively correlated with patient survival, and T cell numbers were negatively correlated to serum IL-6, IL-10, and TNF-*α* concentration [38]. The rapid production of cytokines in large amounts in response to infection is known as a cytokine storm and has been considered an important contributor to Acute Respiratory Distress Syndrome (ARDS) [38]. T cells play a critical role in viral clearance, however overexposure and persistent stimulation of a virus can lead to functional decline inducing T cell exhaustion and loss of cytokine production. T-cell exhaustion has been observed in several cancers as well as in chronic infections such as TB [54]. In LMTBI, exhausted T cells show a progressive loss of secretion of IL-2, IFN-*γ* and TNF-*α*. Cytokines including TNF-*α*, IFN-*γ*, T cells, and upregulation proteins are important for the maintenance of the granuloma in the lung parenchyma, and therefore understanding the pathogenesis of these pathogens individually is crucial to understanding their role in co-infection and how LMTBI may become activated. Immunomodulation in response to each pathogen tends to induce an unbalanced inflammatory response, which can promote the progression and worsening of both diseases.

In a study conducted by Riou *et al.* [55] they found that acute SARS-CoV-2 infection may not immediately result in progression of latent Mtb to subclinical or active TB disease but found a significant reduction of Mtb–specific CD4^+^ T cells in COVID-19 patients. This decline in Mtb-specific CD4^+^ T cells could affect the ability of the host to control latent or new Mtb infection as an intact T cell response is crucial in maintaining the Mtb granuloma [55]. The loss of the structure of the granuloma in LMTB due to cytokine storm and T cell exhaustion is one of several mechanisms that have been proposed regarding the pathogenesis of co-infection and the activation of LMTB as a result of SARS-CoV-2. Additional mechanisms that have been postulated include reactivation due to lung inflammation due to the virus, flaring of tubercular lesions triggering Type I interferon signaling that is also important for mycobacterial growth, and activation of the mesenchymal stem cell mediated defense mechanism [56].

Some have inspected the possibility of unintentional activation of LMTB with anti-inflammatory agents intended to lower inflammation caused by another pathogen. COVID-19 is an inflammatory lung disease, and treatment with anti-inflammatory agents would be a logical step in COVID-19 treatment. Anti-inflammatories, however, do by definition attenuate the pro-inflammatory cytokines responsible for the inflammatory process, including TNF-*α* and IFN-*γ*, which are two cytokines responsible in the defense mechanisms of both TB and COVID-19 [11]. Without defenses such as these, it has been argued that activation of LMTB will occur due to the dampening effects of the immune system [16,57], however more research is required to come to a definitive conclusion [42].

## 7. Effect of Covid on Active TB

Both COVID-19 and TB are primarily respiratory illnesses that can be transmitted in the air, eliciting hyperinflammatory state in the lung. It is thus reasonable to speculate that the hyperinflammatory milieu induced by COVID-19 could accelerate TB disease progression and vice versa [56]. A major concern of co-infection with active TB in addition to poorer treatment outcomes is the potential for overlooking diagnosis of TB due to overlapping clinical features. It is also important to consider the prevalence of drag resistant strains of TB in co-infections. More studies are currently required, but it has been previously observed that variables related to mortality in DR-TB probably do not differ dramatically from those of the general population [58].

A case series of 7 patients with co-infection in Saudi Arabia was reported with each case showing signs of TB prior to confirmation of co-infection through microbiological testing [59]. Although the patients had differing presentations of disease, they were all found to have atypical imaging characteristics for COVID-19 that raised suspicion for alternative pulmonary disease. While COVID-19 is likely to show a bilateral, diffuse, infiltrative pattern, TB is likely to show a unilateral upper-zone opacity. Immune mediators and pathways that perhaps drive necrosis and cavitation during TB may lead to subsequent fibrosis [60] and affect the function of the respiratory tract, increasing the risk of pneumonia and respiratory failure. All patients demonstrated improved outcomes except for one 30-year-old female with no known prior medical conditions who passed away due to extensive disease. Another case series reported 4 migrant workers residing in dormitories in Singapore who were co-infected and also had atypical radiographic features [61]. A case report of a 28-year-old man described co-infection with COVID-19 with lung infiltrates and disseminated TB, with lesions found in the liver and a tuberculoma excised from the brain [62].

While many co-infection cases have shown good outcomes with treatment, fatal cases of co-infection have been reported. One reported fatal case is of a 38-year-old-male without significant medical history who presented with symptoms of low-grade fever, cough with expectorations, and shortness of breath for a month and a half; SARS-CoV-2 testing was positive through Reverse Transcription- Polymerase Chain Reaction (RT-PCR) [63]. Even with treatment including IV antibiotics, hydroxychloroquine sulfate, and oxygen supplementation, the patient remained symptomatic. With prolonged and untreated symptoms, sputum samples for TB were sent. Despite empirical treatment for TB, the patient rapidly deteriorated and died: diagnosis of TB was confirmed post mortem [63]. An additional fatal case of coinfection has been reported in a 3-month-old infant in Botswana who presented with fever and respiratory distress in the setting of failure to thrive [64]. The patient’s rapid clinical decline could not be explained by TB alone, and presence of diffuse microthrombi on autopsy together suggested a likely synergistic pathophysiologic effect of co-infection. A study by Sy *et al.* [65] explored co-infection cases in the Philippines, comparing risk of death and recovery in COVID-19 patients with and without TB. They found that patients with TB had 2.17 times higher risk of death than those without and risk of recovery in patients with TB was 25% lower than in those without [65]. The final matched sample consisted of 530 patients, with 106 cases with TB. Majority of cases were male patients with hypertension and/or DM II and most of the deaths seen were among older individuals with several comorbidities. Their results are consistent with an additional preliminary cohort study conducted by Davies which concluded COVID-19 patients with a current TB diagnosis had a 2.5 times higher risk of death, and those with previous TB had a 50% higher risk [66].

## 8. Management of Coinfection Condition

Age, poverty, malnutrition, and co-morbidities such as TB indicate higher risk of more severe COVID-19 [33]. Low income populations have a tendency to become more vulnerable to infectious disease due to several factors including reduced access to adequate healthcare and suboptimal living conditions. Moreover, coinfection with both tuberculosis and COVID-19 is of special concern to these populations where tuberculosis is already a risk of higher significance.

The pandemic affected efforts to eradicate tuberculosis. Consequently, there has been an 18% decline in newly diagnosed TB cases and an increase in TB deaths, which is speculated to be a side effect of recent lack of access to diagnosis and treatment resources for TB. In an effort to better fund COVID-19 eradication efforts, many countries have reduced their resources allotted to other healthcare objectives such as the infrastructure in place to combat TB [1]. In addition to a reduced emphasis on TB healthcare, symptomatic patients may have been reluctant to seek medical attention leading to a delay or lack of active TB diagnosis and thus worsening of their condition [67]. Asymptomatic individuals who have had known contact with TB patients may delay seeking medical attention during the COVID-19 pandemic, thus leading to reduced diagnosis of LMTBI as well.

## 9. Summary

There are many reasons why a host’s immune system may be rendered inadequate enough to maintain granulomatous retention of LTBI and thus cause activated TB. This review compares the characteristics of different immune signaling molecules in the specific context of COVID-19 and whether co-infection with LTBI or active TB can lead to more deadly complications, whether it be because of the co-infection itself or the management, or lack thereof, of the co-infection due to lack of knowledge of the condition or healthcare infrastructure changes due to the pandemic.

IL-10 levels will see an increase during TB infection. Some of its key functions in TB are to suppress phagosomal maturation and nitrous oxide production, which are both functions that do not aid in the suppression of the disease. IL-10 can therefore aid in the reactivation of TB and disease progression. In COVID-19 infection, it is seen to be upregulated during a cytokine storm and a consistent correlation between disease severity and IL-10 levels has been observed.

TNF-*α*. is a pro-inflammatory molecule: during TB infection, it will cause an increased production of IL-1 and PGE2, which ultimately leads to its inflammatory effects. In COVID-19 infection, it has been observed to play a key role in response against the virus and is seen to be upregulated during a cytokine storm.

IFN-*γ*, the only class II IFN, works through the activation of the JAK-STAT pathway to convey its effects. In a TB infection, it works to increase antigen presentation in Antigen Presenting Cells (APCs) and upregulates lysosomal activity in macrophages, causing an enhanced killing of TB mycobacteria. It also works to upregulate MHC I/MHC II and leukocyte migration during TB infection. During COVID-19 infection, its key role in the response against the virus is the activation of the cytokine storm through JAK-STAT-activated NF-kB signaling. Class I and III IFN groups mainly work in a TB infection as pro-inflammatory molecules. In a COVID-19 infection, they have a very different role. They cause a very diverse viral inhibition mechanism at almost every step of viral replication in the host cell.

TGF-*β* is an anti-inflammatory cytokine responsible for down regulating activity of cytotoxic and helper T cells during infection and an upregulation in Treg cells. TB infection will generally see an increase in TGF-*β*. In COVID-19 infection, it is a significant contributor to symptoms through the activation of apoptosis, inflammation, and lung fibrosis.

Not every molecule in this article has been found to have significant actions taken during both TB infection and COVID-19 infection. ACE-2 does not appear to have a significant contribution to TB infection, but is the host receptor for the COVID virus’ Spike protein. In TB infection, iTreg cells work locally to mediate suppressive effects through the use of IL-10, IL-35, and TGF-*β*. In COVID-19 infection, it has been considered to be a first line of defense against unregulated inflammatory response, but research has not shown a consistent correlation between severity of disease and number of T-reg cells during host response. IL-35 is responsible for upregulation of T-reg cells and downregulation of Th1 and Th17 cells during TB infection. Significant research on the actions of IL-35 during COVID-19 infection has not yet been discovered.

## 10. Conclusions

Currently there is no available experimental data on immunopathological effects of TB/COVID-19 co-infection. However, given our current understanding of the etiology of TB and COVID-19 independently as well as reviewing current literature on co-infection, we can postulate the interactions of these airborne diseases with co-infection. A thorough understanding of the immunological and clinical similarities and differences of these pathogens and further investigation of their interactions may lead to a hopeful improved system of therapeutic strategies for co-infection as well as improved prevention measures. Several studies explored in this review have suggested a synergistic or additive effect when Mtb and SARS-CoV-2 share the same host, leading to increased severity of disease, especially if presenting with comorbidities such as DM II, HTN, and obesity.

There are several mechanisms that have been proposed that may explain why co-infection between TB and COVID-19 causes a synergistic effect. One popular proposed mechanism involves the combined effect of TB and COVID-19 infection likely causing a pronounced lymphocytopenia and consequently a CD4^+^ cell decrease due to exhaustion of these immune cells. This disrupts regulation of the immune response against these pathogens, ultimately leading to an exacerbation of pro-inflammatory cytokines. The resulting cytokine storm as the additive effect of active cells in the lung leads to immunopathology, increasing the risk of morbidity and mortality. Several publications in this review have described the implications of co-infection on the lung parenchyma. As immune mediators cause necrosis and cavitation during TB infection and may lead to subsequent fibrosis and affect the function of the respiratory tract, this is a likely explanation for why patients with active TB have a worse prognosis with SARS-CoV-2 co-infection as seen in the current available reviewed literature.

COVID-19 is an inflammatory lung disease, and it follows that treatment with anti-inflammatory agents would be a logical step in COVID-19 treatment. Therefore, the use of immunomodulators should be carefully considered in individuals given their clinical history, especially in areas with high TB incidence as the use of blocking pro-inflammatory cytokines that maintain the structural integrity of the granuloma may lead to activation of latent TB. Understanding how pathogens interact with the same host pulmonary microenvironment and the biomolecular effects of these contributors to pathogenicity is fundamental for prevention and treatment of TB/COVID-19 co-infection. A summary of these contributors to pathogenicity of TB and COVID-19 has been summarized in Table 1. As great effort and focus is placed into stopping the ongoing COVID-19 pandemic globally, it is of utmost importance to not neglect TB, one of the oldest and deadliest infectious diseases of mankind which is both preventable and treatable.

Our search was conducted through the PubMed database. Our search criteria included the following keywords, both combined and separated: “COVID-19”, “Latent TB”, “Active TB”, “Tuberculosis”, “SARS”, “MERS”, “immune response”, “anti-inflammatory agents”, “epidemiology”, “bronchiectasis”, “fibrosis”, “cavitation”, “TB treatment”, “IL-10”, “TNF-*α*”, “IFN”, “TGF-*β*”, “IL-35”, “Regulatory T cells” and “co-infection”.

## Figures and Tables

**Table 1.** Comparison between various contributors of disease in TB and COVID-19, respectively, based on discussed material in this article.

Cytokines and modulators	Mycobacterium tuberculosis	SARS-CoV-2
	• Upregulated during TB infection	• Upregulated during cytokine storm
IL-10	• Suppress phagosomal maturation, suppress nitrous oxide production	• Consistent correlation between disease severity and IL-10 levels
	• Can drive TB reactivation and disease progresssion	

TNF-*α*	• Upregulated production of IL-1 and PGE2→inflammation	• Key role in response against virus
		• Upregulated during cytokine storm

IFN-*γ* (Class II IFN)	• Activates JAK-STAT→more antigen presentation and lysosomal activity in macrophages	• Key role in response against virus
	• Upregulates MHC I/MHC II and leukocyte migration	• Activate JAK/STATĉytokine storm
	• Upregulates macrophage-activated killing of TB	

Class I and III IFN	• Bind opioid receptors PGE2→inflammation	• Diverse viral inhibition mechanisms at almost every step of viral cycle

TGF-*β*	• Downregulate activity of cytotoxic and helper T cells	• Significant contributor to COVID-19 symptoms
	• Differentiation of Treg from naïve CD4^+^ cells	
	• Downregulate inflammation	• Activates apoptosis, inflammation, lung fibrosis
	• Upregulated in number during TB infection		

ACE-2	• No significant contribution currently found	• Receptor for Spike protein
		• Helps attenuate inflammatory lung disease

Treg	• tTreg: systemic homeostasis and self-tolerance	• First line of defense against unregulated inflammatory response
	• iTreg: works locally: uses cytokine IL-10, IL-35 and TGF-*β* to mediate suppressive effects	• Inconsistent response during COVID-19 infection

IL-35	• Downregulate development of Th1 and Th17	• No significant contribution currently found
	• Induces T-reg proliferation	

## Data Availability

Data sharing not applicable. No new data were created or analyzed in this study. Data sharing is not applicable to this article.

## Abbreviations

LTBI: Latent Tuberculosis Infection
TB: Tuberculosis
Mtb: Mycobacterium tuberculosis
SARS-CoV-2: Severe Acute Respiratory Syndrome Coronavirus 2
COVID-19: Coronavirus Disease-19
IL: Interleukin
TNF: Tumor Necrosis Factor
IFN: Interferon
TGF: Transforming Growth Factor
Treg: Regulatory T Cells
DR-TB: Drug-Resistant tuberculosis
MDR/RR-TB: Multidrug and rifampicin-resistant Tuberculosis
XDR-TB: Extensively Drug Resistant TB
MERS: Middle East Respiratory Syndrome
EAA1: Early Endosomal Autoantigen 1
PGE2: Prostaglandin E2
ISG: Interferon-Stimulated Genes
Th: Helper T cell
RT-PCR: Reverse Transcription Polymerase Chain Reaction
HIV: Human Immunodeficiency Virus
iTreg: Induced Regulatory T Cells
tTreg: Thymic Regulatory T Cells
IT1: Inducible T Regulatory Type 1 Cells
DMII: Diabetes Mellitus Type 2
CVD: Cardiovascular Disease
COPD: Chronic Obstructive Pulmonary Disease
ACE: Angiotensin Converting Enzyme
PAMP: Pathogen Associated Molecular Protein
PRR: Pattern Recognition Receptor
DAD: Diffuse Alveolar Damage
